# EUS-guided ablation for pancreatic cystic lesions: An updated review

**DOI:** 10.1097/eus.0000000000000144

**Published:** 2025-11-03

**Authors:** Mengruo Jiang, Lisi Peng, Yuwei Sun, Shiyu Li, Zhaoshen Li, Liqi Sun, Haojie Huang, Jin Zhendong

**Affiliations:** 1 Department of Gastroenterology, National Clinical Research Center for Digestive Diseases, Changhai Hospital, Navy Medical University, Shanghai, China; 2 Department of Gastroenterology, Sir Run Run Shaw Hospital, Zhejiang University School of Medicine, Hangzhou, Zhejiang Province, China; 3 National Key Laboratory of Immunity and Inflammation, Naval Medical University, Shanghai, China; 4 Department of Gastroenterology, 72nd Group Army Hospital, Huzhou University, Huzhou, Zhejiang Province, China.

**Keywords:** EUS-guided pancreatic cyst chemoablation, EUS-guided radiofrequency ablation, Ethanol, Lauromacrogol, Paclitaxel, Pancreatic cystic lesions

## Abstract

Pancreatic cystic lesions (PCLs) are a globally prevalent condition, with incidence increasing with age. The proper treatment modality for PCLs remains a controversial topic. EUS-guided ablation represents an innovative and promising minimally invasive treatment for selected PCL patients, including EUS-guided pancreatic cyst chemoablation (EUS-PCC, such as ethanol, lauromacrogol, chemotherapeutic agents, and combination therapies) and EUS-guided radiofrequency ablation (EUS-RFA). Each treatment modality varies in treatment indications, efficacy, and safety. Herein, with ongoing advancements in clinical research, we present a comprehensive, updated review of procedural techniques, patient selection, clinical outcomes, and adverse events for EUS-PCC and EUS-RFA.

## INTRODUCTION

Pancreatic cystic lesions (PCLs) are a heterogeneous group of tumors with distinct biological features, characterized by a cystic cavity formed by pancreatic epithelial and/or interstitial tissues.^[1]^ Recently, incidental PCLs in asymptomatic patients have increasingly been identified.^[2]^ In 2024, a proportional meta-analysis of 15 studies involving 65,607 subjects showed that the global prevalence of PCLs ranges from 13% to 18%, with prevalence increasing with age.^[3]^

Among all PCLs, intraductal papillary mucinous neoplasms (IPMNs), mucinous cystic neoplasms (MCNs), and solid pseudopapillary neoplasms have the potential to progress into invasive carcinoma.^[4,5]^ Over the past decades, the management of PCLs has remained a controversial topic.^[1,6]^ Guidelines for managing PCLs recommend surgical resection for symptomatic cysts, cysts with high-risk features, and cysts with definitive malignant pathological outcomes.^[7,8]^ However, given that these high-risk features are not entirely synonymous with malignancy and the incidence of pancreatic surgery–associated AEs is high (20%–40% for severe AEs and 1%–5% for mortality),^[9,10]^ the difficult clinical decision between radiographic surveillance and surgical resection has been proposed. It is estimated that up to $3.6 million is spent on surveillance for each PCL-related cancer detected in the United States, with no meaningful impact on PCL-related cancer mortality.^[11,12]^ Furthermore, ongoing radiographic surveillance is still required after surgery. Therefore, alternative treatments with a less invasive nature are urgently needed to balance the risks of surveillance and surgical resection.

Recently, EUS-guided pancreatic cyst ablation (EUS-PCA), as a derivative technique of EUS-guided fine-needle aspiration (EUS-FNA), has gradually emerged as an innovative and promising minimally invasive treatment for appropriately selected PCLs.^[13–15]^ Recent studies on EUS-PCA have provided favorable results. In 2024, Moyer et al.^[10]^ reported the long-term follow-up results of 2 randomized, prospective clinical trials on chemotherapy for ablation and resolution of mucinous pancreatic cysts (ChARM). The prospective study, which included 52 patients, demonstrated that the medical costs for radiological surveillance following ChARM treatment were $7200.00, compared with $19,437.44 and $12,526.52 for patients treated and monitored according to the Fukuda and ACG guidelines, respectively. Furthermore, no recurrence or development of cyst-related malignancies occurred during a 5-year follow-up, which indicated that the frequency of radiological surveillance can be reduced. Additionally, a propensity score–matched (PSM) analysis assessing the clinical benefits of EUS-PCA compared with the natural course of PCLs indicated that the surgical resection rate in the EUS-PCA group was significantly lower than that in the natural course group (4.8% *vs.* 26.2%; *P* < 0.001).^[16]^ Furthermore, a recent comparative study by Cho et al.^[17]^ performed 1:1 PSM for patients with unilocular or oligolocular PCLs who underwent either EUS-PCA or surgical treatment. The retrospective study, which included 620 patients, revealed that the EUS-PCA group had a lower 10-year cumulative incidence of long-term complications (1.6% *vs.* 33.5%; *P* = 0.001), readmission (1.0% *vs.* 15.2%; *P* = 0.001), and diabetes (2.2% *vs.* 22.8%; *P* = 0.001). The authors concluded that, for selected PCL patients, EUS-PCA offers superior long-term safety and pancreatic function preservation compared with surgical resection.

**Figure 1:**
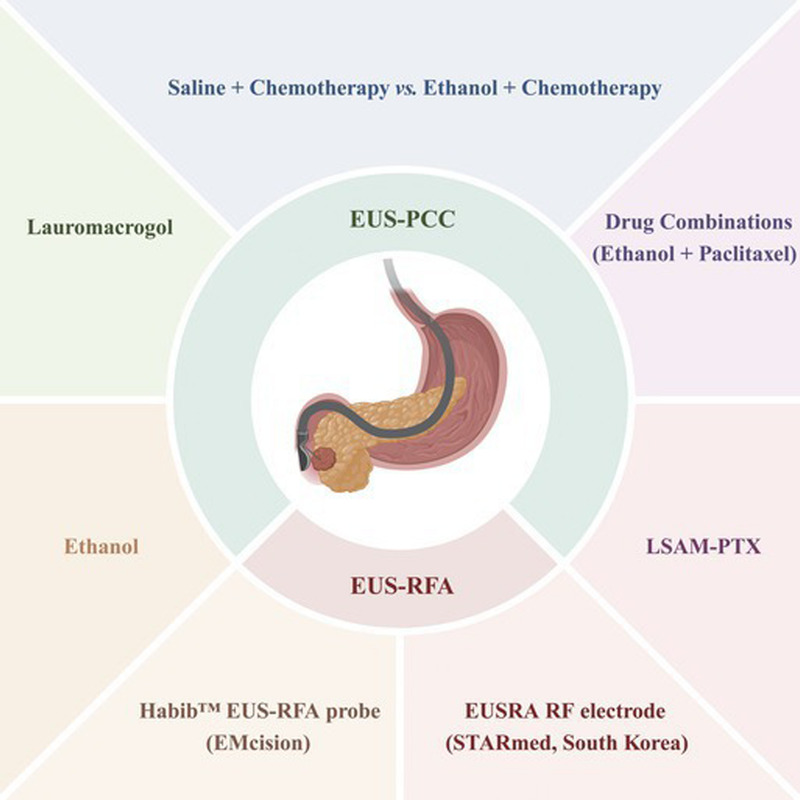
EUS-guided ablation for pancreatic cystic lesions. EUS-PCC: EUS-guided pancreatic cyst chemoablation; EUS-RFA: EUS-guided radiofrequency ablation.

These high-quality clinical studies establish the role of EUS-PCA in the management of PCLs. Therefore, we conducted a comprehensive updated review to summarize the studies that evaluate the efficacy and safety of EUS-PCA, including EUS-guided pancreatic cyst chemoablation (EUS-PCC) and EUS-guided radiofrequency ablation (EUS-RFA) [Figure 1].

## EUS-GUIDED PANCREATIC CYST CHEMOABLATION

EUS-PCC is a minimally invasive technique based on EUS-FNA to precisely deliver ablative agents to target tissues. Recent studies suggest that the agent should be ethanol, lauromacrogol, or combination therapies such as ethanol-paclitaxel regimens. Therapeutic outcomes vary significantly based on the specific agent and protocol [Table 1].

**Table 1:** Clinical studies on EUS-guided pancreatic cyst chemoablation for pancreatic cystic lesions.

References	Study design	Comparison	No. ofpatients	Type of PCLs, *n*	Mean/median diameter (mm)	Mean/median follow-up (mo)	Outcomes*		Adverse events, *n*
							CR, *n* (%)	PR, *n* (%)	
**Ethanol**									
Gan et al., 2005	Prospective	—	25	MCN (14), IPMN (3), SCN (3), indeterminate (2), PC (1)	19.4	12.0	8 (35)	2 (9)	0
DeWitt et al., 2009	Prospective	Ethanol *vs.* saline	25 *vs.* 17	MCN (17), IPMN (17), SCN (5), PC (3)	21.6 *vs.* 23.6	3–4 mo after the first lavage	Reduction in mean cyst surface area* (−42.9% *vs.* −11.4%)		Abdominal pain (5), pancreatitis (2), intracystic bleeding (1) *vs.* abdominal pain (3)
DiMaio et al., 2009	Retrospective	Baseline *vs.* 1 lavage/2 lavages	13	IPMN (13)	20.1 *vs.* 17.0/12.8	3–6 mo after the lavage	0 (0) *vs.* 0 (0) /5 (38)	—	Abdominal pain (1) after the first lavage and abdominal pain (1) after the second lavage
DeWitt et al., 2010	Prospective	—	12	MCN (9), IPMN (1), SCN (1), PC (1)	18.0	26 mo after the initial cyst resolution	No evidence of cyst recurrence in any of the patients*		Abdominal pain (4), pancreatitis (2)
Caillol et al., 2012	Retrospective	—	13	MCN (13)	24.0	26.0	11 (85)	—	0
Park et al., 2016	Prospective	—	91	SCN (33), indeterminate (28), MCN (12), IPMN (9), PC (9)	30.0	40.0	41 (45)	37 (41)	Abdominal pain (18), fever (8), pancreatitis (3)
Gómez et al., 2016	Prospective	—	23	IPMN (15), MCN (4), nonmucinous cysts (4)	27.5	40.0	2 (9)	—	Abdominal pain (1), pancreatitis (1)
Choi et al., 2018	Retrospective	Ethanol lavage *vs.* natural course	84 *vs.* 84	—	23.1 *vs.* 23.0	78.9 *vs.* 75.9	Surgical resection rate: 4.8% *vs.* 26.2%* (*P* < 0.001)		—
Jang et al., 2019	Retrospective	—	8	IPMN (8)	—	18.2	Patients with IPMNs exhibit malignant conversion after EUS-PCA*		—
**Lauromacrogol**									
Linghu et al., 2017	Prospective	—	29	MCN (15), SCN (12), indeterminate (2)	28.6	9.0	11 (38)	9 (31)	Pancreatitis (2), fever (1)
Du et al., 2022	Prospective	—	70	SCN (34), MCN (27), indeterminate (9)	35.5	15.0	26 (47)	15 (27)	Abdominal pain (3)
Gao et al., 2025	Prospective	1% *vs.* 2% *vs.* 3% lauromacrogol	15 *vs.* 12 *vs.* 15	SCN (22), MCN (20)	26.2 *vs.* 32.9 *vs.* 28.5	8.3	1 (7) *vs.* 0 (0) *vs.* 7 (70)	—	0 *vs.* 0 *vs.* Pancreatitis (1)
**Drug Combinations (Ethanol + Paclitaxel)**									
Oh et al., 2008	Prospective	—	14	Indeterminate (6), SCN (3), Lymphangioma (3), MCN (2)	25.5	9.0	11 (79)	2 (14)	Hyperamylasemia (6), pancreatitis (1), abdominal pain (1)
Oh et al., 2009	Prospective	—	10	SCN (4), MCN (3), indeterminate (3)	29.5	8.5	6 (60)	2 (20)	Pancreatitis (1)
Oh et al., 2011	Prospective	—	52	Indeterminate (26), SCN (15), MCN (9), PC (2)	31.8	21.7	29 (62)	6 (13)	Fever (1), abdominal pain (1), pancreatitis (1), splenic vein obliteration (1)
DeWitt et al., 2014	Prospective	—	22	IPMN (12), MCN (6), SCN (4)	25.0	27.0	10 (50)	5 (25)	Abdominal pain (4), pancreatitis (3), peritonitis (1), gastric wall cyst (1)
Choi et al., 2017	Prospective	—	164	MCN (71), indeterminate (63), SCN (16), IPMN (11), PC (3)	32.0	69.0	114 (72)	31 (20)	Fever (1), pancreatitis (6), pseudocyst (2), abscess (2), intracystic hemorrhage (1), pericystic spillage (1), portal vein thrombosis (1), splenic vein obliteration (1), main pancreatic duct stricture (1)
Kim et al., 2017	Prospective	—	36^†^	MCN (16), IPMN (14), SCN (5), PC (1)	25.8	22.3	19 (56)	7 (21)	Abdominal pain (4), pancreatitis (4), intracystic hemorrhage (1)
An et al., 2022	Retrospective	—	12^†^	MCNs (10), SCA (1), epidermoid cyst (1)	37.0	18.0	Diffuse eggshell-like calcification along the pancreatic cystic walls with residual lining epithelia and/or ovarian-type stroma was characteristic of pancreatic cysts after EUS-PCA*		—
Cho et al., 2024	Retrospective	EUS-PCA *vs.* surgery	310^†^ *vs.* 310	MCN (197), indeterminate (117), SCN (70), IPMN (236)	37.2 *vs.* 36.9	66.0 *vs.* 63.6	10-y cumulative long-term morbidity rate*: 1.6% vs 33.5% (*P* = 0.001)		Pericystic spillage (2), pancreatitis (1), pancreatic ductal stricture (1) *vs.* Postoperative fluid collection or postoperative pancreatic fistula (18), bleeding (7), anastomotic stricture (12), liver abscess (2), wound dehiscence (1), foreign body (1), wound infection (1), incisional hernia (1), a-loop syndrome (1)
**Improvement of drug combinations (saline + paclitaxel and gemcitabine *vs.* ethanol + paclitaxel and gemcitabine)**									
Moyer et al., 2016	Prospective	Saline + paclitaxel and gemcitabine *vs.* ethanol + paclitaxel and gemcitabine	6 *vs.* 4	MCN (7), IPMN (2), indeterminate (1)	30.0	12.0	4 (67) *vs.* 3 (75)	—	0 *vs.* Pancreatitis (1)
Moyer et al., 2017	Prospective	Saline + paclitaxel and gemcitabine *vs.* ethanol + paclitaxel and gemcitabine	21 *vs.* 18	IPMN (27), MCN (9), indeterminate (3)	25.0	12.0	14 (67) *vs.* 11 (61)	—	0 *vs.* Pancreatitis (1), abdominal pain (4)
Moyer et al., 2024	Prospective	—	52**^‡^**	IPMN (36), MCN (9), indeterminate (7)	28.7	63 mo for 36 ChARM patients and 24 mo for 24 ChARM II patients	36 (69)	11 (21)	—
**LSAM-PTX**									
Othman et al., 2022	Prospective	—	19	IPMN (17), MCN (2)	28.5	6.0	Cyst volume reduction* (10%–78%) was seen in 12 (70.6%) patients.		Abdominal discomfort (6.1%), peripheral edema (6.1%), nausea (6.1%), headache (5.1%), fatigue (5.1%)
Krishna et al., 2024	Prospective	—	5	IPMN (5)	31.8	31.0	Mean cyst volume reduction*: 9.6 ± 5.1 to 2.2 ± 1.1 mL (*P* = 0.016)		Pancreatitis (1)

*While complete or partial resolution rates were not reported in the article, other indicators were used to describe the outcomes.

**^†^**Patients underwent EUS-PCA, either with ethanol alone or with a combination of ethanol and paclitaxel. Because the 2 groups could not be separated in the subsequent data analysis, the results are presented as a whole.

**^‡^**Fifty-two patients (in the ChARM trial or ChARM II trial) received either ethanol or saline lavage, followed by EUS-guided chemoablation using a chemotherapeutic mixture of paclitaxel and gemcitabine.

−: Not reported in the original article; PCLs: Pancreatic cystic lesions; CR: Complete resolution; PR: Partial resolution; IPMN: Intraductal papillary mucinous neoplasms; SCN: Serous cystic neoplasm; MCN: Mucinous cystic neoplasm; PC: Pseudocyst; EUS-PCA: EUS-guided pancreatic cyst ablation.

### Procedural technique

Before EUS-PCC, linear EUS is used to comprehensively evaluate the pancreatic cyst. For cysts with a diameter of 2 to 3 cm, a 22-gauge needle is used for aspiration. For cysts larger than 3 cm or those with a known history of high mucinous cystic fluid, a 19-gauge FNA needle is used. The FNA needle is passed transgastrically or transduodenally into the center of the cyst, after which a syringe is attached to the proximal end of the needle to carefully evacuate the cystic content.^[17]^ The collected fluid can be further analyzed to assist in the diagnosis of the pancreatic cyst. If the cystic fluid is too viscous to drain sufficiently, saline can be injected to reduce its viscosity. A small amount of fluid should remain around the needle tip to prevent the complete collapse of the cyst. The puncture needle should remain in the cyst prior to the injection of the ablative agent to avoid injury to the pancreatic duct wall and prevent extravasation of the agent. Under ultrasound monitoring, an equal amount of ablative agent is injected into the cyst, followed by lavage of the cyst for 3 to 5 minutes, alternating between filling and draining the cavity. For ethanol, it is essential to drain as much of the ethanol mixture as possible from the cyst following ethanol lavage.^[18]^ Chemotherapy drugs, such as paclitaxel, are typically injected into the cyst cavity after ethanol lavage, where they can remain and exert a long-term therapeutic effect.^[19]^ Regarding lauromacrogol, one-half to two-thirds are removed after lavage, whereas the remaining one-third to one-half is retained within the cyst to further enhance therapeutic efficacy.^[14,20]^ Once the injection and lavage are completed, the needle is removed from the cyst. According to American Society for Gastrointestinal Endoscopy guidelines, prophylactic antibiotics may be administered to patients.^[21]^

### Patient selection

It is important to note that chemoablation is not suitable for all patients, and it benefits only a specific subset of patients with PCLs.^[5,22]^ The most recent updates on the patient populations suitable for chemoablation suggest that pancreatic cysts with a diameter between 2 and 6 cm, including mucinous cysts and uncertain cysts, are eligible.^[23]^ In contrast, lesions classified as nonmucinous cyst types (such as pseudocysts, simple cysts, and serous cystic neoplasms [SCNs]) are considered contraindications to chemoablation to avoid overtreatment.^[7]^ Moreover, for PCLs with clear malignant features or cytologically suspected malignancy and for patients with a reasonable life expectancy of less than 3 to 5 years, EUS-PCC should not be performed. The relative contraindications to EUS-PCC are cysts with the following high-risk features^[23]^: main pancreatic duct dilation >5 mm, epithelial wall nodules >3 to 5 mm, thickened walls or septa >3 mm, obstructive signs in the common bile duct or pancreatic duct, or lymphadenopathy associated with the cyst. Treatment decisions for patients with these relative contraindications should be evaluated by a multidisciplinary team.

### Ablative agents

#### Ethanol

Ethanol, the first ablative agent described for EUS-PCC,^[18]^ offers several advantages, including widespread availability, low cost, ease of administration, and rapid ablation. It is believed to induce tissue necrosis by rapidly dehydrating and fixing the target tissue, resulting in protein denaturation, endothelial cell destruction, and local thrombosis. Additionally, the damage to target cells caused by ethanol triggers inflammatory cell infiltration and fibroblast proliferation, ultimately leading to the inactivation of the local tissue.^[24,25]^

In 2005, Gan et al.^[18]^ pioneered the exploration of ethanol ablation for the treatment of PCL with 25 patients included. The results revealed the safety of ethanol lavage for PCLs, with 8 patients (35%) showing complete resolution (CR) on imaging follow-up. The feasibility of ethanol ablation was also validated in a study by Caillol et al.,^[26]^ and CR was achieved in 85% of the 13 mucinous cysts. The authors attributed the higher ablation rate to the smaller size of the cysts. Similarly, Oh et al.^[27]^ and Park et al.^[28]^ also reported that cyst size was a predictor of CR. To improve the efficacy of ethanol ablation, DiMaio et al.^[29]^ conducted a retrospective analysis of 13 patients who underwent 2 or more EUS-guided ethanol ablation procedures. Their findings revealed that, compared with the baseline mean cyst surface area and maximum diameter (5734 ± 6846 mm^2^; 20.1 ± 7.1 mm), 2 ethanol lavage treatments (2311 ± 4093 mm^2^, *P* = 0.008; 12.8 ± 9.6 mm, *P* = 0.0002) resulted in a significantly greater reduction in cyst surface area and size than a single ethanol lavage (4906 ± 9240 mm^2^, *P* = 0.52; 17.0 ± 9.8 mm, *P* = 0.06), suggesting the added effectiveness of booster ablation. To further assess the long-term durability of successful cyst resolution following EUS-guided ethanol lavage, DeWitt et al.^[30]^ conducted a prospective cohort study, and the results indicated that none of the patients showed evidence of cyst recurrence on follow-up CT scans, suggesting that the long-term outcomes of ethanol ablation are stable. Furthermore, short-term follow-up results in a prospective, multicenter, randomized trial revealed that, compared with the saline lavage group, ethanol lavage resulted in a significantly greater reduction in mean cyst surface area (−42.9% *vs.* −11.4%; *P* = 0.009).^[24]^

Due to the biological heterogeneity of PCLs, it must be acknowledged that not all patients will benefit from ethanol ablation. Some studies have shown that ethanol ablation offers limited therapeutic efficacy for IPMNs.^[28,31]^ Park et al.^[28]^ reported that the CR rate of ethanol lavage varied significantly across different types of PCLs: SCNs, 58%; MCNs, 50%; IPMNs, 11%; and uncategorized cysts, 39% (*P* < 0.0001). The results of this prospective study demonstrated that IPMNs had the poorest response to ethanol ablation, and this may be related to the mucous material lining the inner wall of the PCLs, as well as the papillary growth pattern and communication with the main pancreatic duct. Moreover, Jang et al.^[31]^ reported 8 cases of malignant transformation after EUS-PCA in IPMN patients. All patients had at least one high-risk feature during ethanol ablation, such as cyst size >3 cm, pancreatic duct dilation, or wall nodules (+). The mean time from EUS-PCA to the detection of malignancy was only 18.2 months, and 2 patients developed tumor spillage or peritoneal seeding after the procedure. Similarly, a single-center, prospective, pilot study involving 23 patients with suspected MCNs or branch duct (BD)–IPMNs ≥1 cm in maximum diameter demonstrated that ethanol ablation was not effective in preventing malignancy for these pancreatic cysts.^[32]^ In this study, one participant was diagnosed with pancreatic adenocarcinoma, thought to have arisen from the treated BD-IPMN, 41 months after undergoing ethanol ablation. These findings indicate that ethanol ablation may not be effective enough to treat IPMNs.

In summary, ethanol is a commonly used and effective ablative agent for EUS-PCC, with varying efficacy based on cyst type and size. Repeated treatments can improve its efficacy. However, its effectiveness is limited in treating IPMNs.

#### Lauromacrogol

Lauromacrogol, a mild anesthetic sclerosant, induces vascular damage by altering the surface tension around endothelial cells.^[20]^ It has been widely used in the treatment of bleeding esophageal varices^[33]^ and cystic lesions, such as renal and hepatic cysts.^[34]^ Compared with ethanol, the mild anesthetic properties of lauromacrogol significantly reduce perioperative pain in patients undergoing EUS-PCC, which has contributed to its increasing attention in recent years.^[14,20]^

Linghu et al.^[20]^ conducted a prospective single-center study involving 29 PCL patients undergoing EUS-guided lauromacrogol ablation. The results demonstrated CR in 11 patients (37.9%) and partial resolution (PR) in 9 patients (31.0%), with no reports of moderate or severe abdominal pain. Only 3 AEs occurred: 2 mild pancreatitis cases and 1 moderate fever. However, the follow-up time was insufficient.^[35]^ A subsequent study by them enrolled 70 suspected PCL patients to further investigate the long-term efficacy.^[14]^ In the 35 patients monitored for a minimum of 12 months, 18 (51.4%) achieved CR, and 9 (25.7%) achieved PR. Remarkably, none of the patients experienced moderate to severe postoperative abdominal pain. In 2025, a triple-blind randomized controlled trial involving 42 patients with PCNs further compared the ablation efficacy and safety of 1%, 2%, and 3% lauromacrogol.^[36]^ The results showed median ablation rates of 94.1%, 82.0%, and 100.0% in the 3 groups, respectively, suggesting that 3% lauromacrogol offers the best efficacy for treating PCNs.

In conclusion, compared with ethanol, lauromacrogol significantly reduces perioperative pain. Nevertheless, further research involving larger sample sizes and extended follow-up periods is warranted.

#### Drug combinations

The combined use of drugs in EUS-PCA was first reported by Oh et al.,^[37]^ and the results were promising. In this study, 14 patients received EUS-guided ethanol lavage followed by paclitaxel injection (EUS-EP). The researchers hypothesized that preliminary ethanol lavage may induce primary distortion of the epithelial lining, allowing subsequently injected paclitaxel to diffuse into the damaged epithelial cells, thereby exerting a synergistic inhibitory effect on the epithelial lining.^[27]^ The results from this study^[37]^ indicated that 11 patients (78.6%) achieved CR, whereas 2 patients (14.3%) experienced PR. To further investigate the long-term efficacy of EUS-EP, they^[38]^ conducted a subsequent study involving 47 PCL patients. After a median follow-up duration of 21.7 months, CR was observed in 29 patients (61.7%) and PR in 6 patients (12.8%). Additionally, Choi et al.^[39]^ enrolled 164 patients, and the results demonstrated that, among patients who achieved CR after EUS-EP, 98.3% maintained remission at the 6-year follow-up. Moreover, multivariate analysis identified the absence of septations and cyst size smaller than 35 mm as predictive factors for complete cyst ablation.

The presence of septations within the cyst increases the surface area to be ablated and makes it difficult to ensure access to various cyst cavities during the procedure, thus hindering effective ablation. Additionally, ethanol only ablates the epithelial layer of the cyst wall, which may be ineffective in treating complex cysts. Given that chemotherapeutic agents have a longer period of ablative activity and deeper tissue penetration compared with ethanol, a clinical trial was conducted to explore the efficacy of EUS-EP in the treatment of septated PCLs.^[27]^ Among the 10 patients with septate cystic tumors, 6 patients (60%) achieved CR. Furthermore, in a case report by Oh et al.,^[40]^ a second puncture was performed during a single EUS-EP procedure in a thick-walled septate cyst to traverse more cyst cavities. A follow-up CT scan showed a significant reduction in cyst volume to 17.92 mL after 4 months of the procedure, and the authors noted that a second puncture could be considered in the same session of cyst ablation to increase the treatment efficacy.

However, the evidence supporting the efficacy of EUS-EP primarily comes from imaging-based cyst ablation, with limited data on its impact on reducing the risk of malignancy or the degree of epithelial ablation. It is well established that premalignant pancreatic tumors and cysts often exhibit defined DNA mutations and deletions, such as K-ras mutations and loss of p16. To assess the changes in DNA of cystic fluid following EUS-EP, a single-center, prospective study involving 22 patients with suspected benign pancreatic cysts was conducted.^[41]^ The analysis of cystic fluid from 19 patients 3 months after EUS-EP revealed that mutations were eliminated in 8 patients who had baseline cystic DNA mutations, whereas 3 patients developed new mutations. The clinical significance of the loss or acquisition of gene mutations after ablation remains unclear, but the absence of gene mutations, particularly K-ras mutations, may interrupt or reduce the risk of malignant progression. Additionally, Kim et al.^[42]^ evaluated the cytological changes of cystic fluid following EUS-PCA in a study involving 36 PCL patients, in which 8 patients received ethanol lavage and 28 patients received EUS-EP. Follow-up cytology of 34 patients showed that 27% of patients exhibited epithelial hyperplasia, 15% showed a loss or decreased cellular atypia, 24% had an increased appearance of macrophages, and 15% showed inflammatory cells. These findings appear to reflect the ablation-induced damage to the cyst wall epithelium, mobilizing inflammatory cells and macrophages and inducing a localized inflammatory response. However, these cytological changes did not predict the overall imaging response after ablation. As for the data on the degree of epithelial ablation, a retrospective analysis by An et al.^[25]^ reviewed the pathological results of 12 pancreatic cysts surgically resected after EUS-EP. The results showed that among the resected cysts, 8 cases (67%) exhibited no or only minimal residual lining epithelial cells. Additionally, the pancreatic cysts presented with diffuse eggshell-like calcification along the cyst wall, and focal residual epithelial lining and/or ovarian-type stroma may represent histopathological features characteristic of PCLs following ablation treatment.

In summary, EUS-EP has shown favorable therapeutic outcomes in managing PCLs, with a significant proportion of patients achieving CR or PR. Notably, this approach is effective even in complex septated cysts. Genomic and cytological analyses of the cysts following EUS-EP reveal its ability to eliminate oncogenic driver mutations, such as K-ras, which may help reduce the risk of malignant transformation.

#### Chemoablation protocol improvement

Previous studies have reported that almost all severe AEs following EUS-guided chemoablation (such as pancreatitis, adjacent venous thrombosis, and peritonitis) are considered secondary to alcohol extravasation or the excessive inflammatory effects of alcohol on surrounding tissues.^[43,44]^ The high incidence of AEs has also hindered the acceptance of EUS-PCC. Therefore, to improve the safety of chemoablation, Moyer et al.^[45]^ conducted a prospective, randomized, double-blind pilot study to compare 2 protocols: (saline lavage + chemotherapy) versus (ethanol lavage + chemotherapy). A chemotherapy cocktail (paclitaxel + gemcitabine) specifically tailored for PCLs was used to enhance the efficacy of ablation. Moreover, when gemcitabine is reconstituted with saline, its low viscosity makes it an ideal second agent for diluting paclitaxel. In the pilot study, 10 MCN patients were randomly assigned to either the alcohol-free or alcohol treatment group,^[45]^ and 67% of patients in the alcohol-free group achieved CR at both 6 and 12 months, whereas the alcohol group had CR rates of 50% at 6 months and 75% at 12 months. One patient (20%) in the alcohol group developed acute pancreatitis, whereas no AEs occurred in the alcohol-free group. Furthermore, a subsequent noninferiority study by Moyer et al.,^[19]^ enrolling 39 MCN patients, showed that at the 12-month follow-up, 67% of patients who underwent the alcohol-free protocol achieved CR, compared with 61% in the alcohol group. In the alcohol group, 6% of patients experienced severe AEs, and 22% experienced mild AEs, whereas no AEs were reported in the alcohol-free group (*P* = 0.01). This suggests that when using a chemotherapy cocktail specifically tailored for PCLs, alcohol is not required for effective ablation, and the removal of alcohol may decrease the incidence of complications. However, the authors noted that the study has several limitations, such as being limited to a single ablation, which might be overly conservative, and the restriction on the amount of chemotherapeutic cocktail used might limit the efficacy in larger cystic tumors. Additionally, these limitations will be addressed in an upcoming multicenter trial.

Recently, to overcome the limitations of short-term, intermittent systemic exposure to cytotoxic drugs when paclitaxel is injected into lesions, a prospective, multicenter, open-label clinical trial was conducted.^[15]^ This trial utilized an innovative, particle-engineered form of paclitaxel, LSAM-PTX, which generates microparticle paclitaxel with a large surface area and prolongs the duration of exposure of the epithelial cyst lining to the paclitaxel particles. The study results demonstrated that injecting LSAM-PTX at a concentration of 15 mg/mL into mucinous PCLs was safe and well tolerated. After 6 months of follow-up, 70.6% of patients exhibited a reduction in cyst volume, with no significant AEs observed. Similar findings were reported in a prospective study conducted by Krishna et al.,^[46]^ which enrolled 5 patients with a total of 6 IPMNs. The study showed that a higher dosing frequency of LSAM-PTX exhibited significant correlations with reductions in cyst volume (*P* = 0.03) and surface area (*P* = 0.04). However, longer-term follow-up and additional research are required to assess the impact of LSAM-PTX on the cyst wall epithelium and on markers associated with cyst destruction and malignant transformation.

In conclusion, ethanol may not be necessary for effective cyst ablation. Additionally, the innovative use of LSAM-PTX in therapeutic protocols also provides new insights into optimizing ablation strategies.

### Adverse events

The high incidence of AEs has limited the broader acceptance of EUS-PCC. However, ongoing research is dedicated to enhancing the safety of this treatment, particularly through alcohol-free protocols^[19,45]^ and the use of LSAM-PTX,^[15]^ as we mentioned above. As research progresses, the safety profile of chemoablation is expected to improve further.

The occurrence of AEs following chemoablation has been a longstanding point of debate among experts, influencing the overall acceptability of this treatment. To comprehensively evaluate the efficacy and safety of EUS-PCA, a meta-analysis involving 15 studies and 840 patients was conducted by Papaefthymiou et al.^[43]^ The results revealed that the overall incidence of AEs postablation was 14%, with the most common being abdominal pain (10.6%), followed by pancreatitis (5.1%). Subgroup analysis indicated that ethanol ablation carried the highest risk of AEs compared with other ablative agents. Moreover, multivariate analysis in a retrospective study conducted by Choi et al.^[44]^ identified suspected intraoperative ethanol leakage (*P* = 0.006) as the strongest predictor of postoperative acute pancreatitis. The direct cytotoxic effects of ethanol on ductal epithelium, inadvertent injection of the ablative agent into the pancreatic parenchyma, or the inflammatory effects of ethanol may be the proposed mechanisms of AEs. Additionally, to assess the systemic effects of ablative agents following EUS-EP, liquid chromatography–tandem mass spectrometry was used to measure plasma paclitaxel concentrations.^[47]^ The results showed that peak plasma paclitaxel concentrations ranged from 0.45 to 14.73 ng/mL, indicating that plasma paclitaxel levels post–EUS-EP were associated with rare systemic adverse reactions. Furthermore, severe postoperative AEs, such as portal vein thrombosis^[48]^ and duodenal stenosis resulting from postprocedural necrotizing pancreatitis,^[49]^ were also described in some case reports.

## EUS-GUIDED RADIOFREQUENCY ABLATION

RFA is a safe and effective treatment modality for focal malignant diseases, using locally thermal-induced coagulative necrosis to ablate dysplastic and neoplastic tissue.^[50]^ RFA generates thermal damage to the target tissue using electromagnetic energy while also stimulating T-cell–mediated immunity by releasing tumor antigens into the bloodstream, thereby enhancing therapeutic efficacy.

Compared with percutaneous and intraoperative RFA approaches, EUS-RFA enables real-time visualization of the probe, facilitating the avoidance of critical structures during penetration, such as major blood vessels, pancreatic ducts, or biliary tracts.^[51]^ This guidance also permits precise procedural planning, reducing the need to traverse normal pancreatic parenchyma and mitigating associated complication risks. Although RFA has been widely utilized in other areas, its application in the pancreas has been restricted due to the inherent thermal sensitivity of pancreatic tissue.^[50]^ However, with advancements in RFA technology, increasing exploration has been dedicated to EUS-RFA for managing PCLs [Table 2].

**Table 2:** Clinical studies of EUS-guided radiofrequency ablation for pancreatic cystic lesions.

References	Study design	No. of PCL patients	Type of PCLs, *n*	Mean/median diameter (mm)	Mean/median follow-up (mo)	Outcomes*		Adverse events, *n*
						CR, *n* (%)	PR, *n* (%)	
Pai et al., 2015	Prospective	6	MCN (4), IPMN (1), microcystic adenoma (1)	36.5	3–6 mo after the procedure	2 (33)	3 (50)	Abdominal pain (2)
Barthet et al., 2019	Prospective	17	IPMN (16), MCN (1)	28.0	12.0	11 (65)	1 (6)	Jejunal perforation (1)
Barthet et al., 2021	Prospective	17	IPMN (16), MCN (1)	29.1	42.6	6 (40)	4 (27)	Jejunal perforation (1), biliary leakage (1)
Oh et al., 2021	Retrospective	13	Microcystic SCN (13)	50.0	9.2	Median volume of the SCN reduction: 37.82 to 10.95 mL*		Abdominal pain (1)
Younis et al., 2022	Prospective	5	IPMNs (4), MCN (1)	36.0	7.0	3 (60)	1 (20)	Abdominal pain (2), pancreatitis (1)
Napoléon et al., 2023	Retrospective	10	IPMN (10)	29.0	—	5 (63)**^†^**	3 (37)**^†^**	—

*While complete or partial resolution rates were not reported in the article, other indicators were used to describe the outcome.

**^†^**Data were missing for 2 of the 10 IPMN patients.

—: Not reported in the original article because the study did not specify relevant data for IPMNs; CR: Complete resolution; PR: Partial resolution; IPMN: Intraductal papillary mucinous neoplasms; SCN: Serous cystic neoplasm; MCN: mucinous cystic neoplasm; PC: Pseudocyst.

### Procedural technique and device

Currently, 4 different EUS-guided radiofrequency (RF) probes are used for pancreatic procedures.^[51]^ Among these, the Habib EUS-RFA probe (EMcision) and the EUSRA RF electrode (STARmed, South Korea) are the most commonly reported in studies for the treatment of PCLs.^[13,52–56]^ The Habib EUS-RFA probe is a monopolar 1F (0.33 mm) electrode, 220 cm in length, which can be inserted through a standard 22-gauge FNA needle. In contrast, the EUSRA RF needle is a 19- or 18-gauge, 140-cm-long electrode that features an integrated internal cooling system, which circulates chilled saline through the needle during the RFA procedure.^[57]^ Both probes are monopolar, with their closed-loop circuit consisting of the RF generator, electrode needle, dispersive electrode (grounding pad), and the patient.^[51]^ The electrode delivers energy to the tissue, creating a high current density and localized heating, whereas the grounding pad completes the electrical circuit.

Prior to the procedure, prophylactic antibiotics and intrarectal nonsteroidal anti-inflammatory drugs should be administered to prevent postoperative infection and pancreatitis.^[58,59]^ The endoscopist then acquires appropriate ultrasonographic images of the target lesion using EUS, evaluating its size, shape, location, and relation to adjacent structures. Color Doppler is activated to avoid major blood vessels, the pancreatic duct, and the bile duct, allowing for the selection of the optimal puncture site. A 19- or 22-gauge puncture needle is then inserted into the lesion, and as much cyst fluid as possible is aspirated from the PCL before RFA. After confirming the position of the needle electrode tip within the lesion’s margins on EUS, RF energy is delivered. During the ablation process, the tissue surrounding the puncture track gradually changes from hypoechoic to hyperechoic, with strong echogenic bubbles indicating successful RFA. The size of the ablation zone varies depending on the wattage, RFA needle tip length, and duration of energy delivery. If multiple RFA sessions are required, the probe can be repositioned to ablate the remaining area of the lesion under EUS guidance.

### Patient selection

Currently, the indications for EUS-RFA in PCLs remain controversial, as no definitive studies have established the criteria for its application. However, emerging evidence suggests that EUS-RFA may demonstrate favorable efficacy in selected premalignant PCLs,^[55]^ with notable ablation effects on mural nodules within cyst walls.^[54,56]^ Compared with ethanol ablation, EUS-RFA appears suitable for targeting IPMNs harboring malignant potential^[53]^ and has also shown promising therapeutic outcomes in microcystic SCNs exhibiting a honeycomb morphology.^[13]^

### Outcomes of EUS-RFA in PCLs

In 2015, a multicenter pilot study enrolled 6 PCL patients (4 MCNs, 1 IPMN, 1 microcystic adenoma) with a mean cystic neoplasm size of 36.5 mm.^[52]^ Follow-up imaging after 3 to 6 months of EUS-RFA revealed CR in 2 patients and a mean 48.4% reduction in cyst size in 3 patients, with AEs occurring in 2 patients due to mild abdominal pain. Preliminary data demonstrated that EUS-RFA was well tolerated for the management of PCLs, with an acceptable safety profile. Similarly, a prospective multicenter open-label phase I study conducted by Barthet et al.^[53]^ involved 17 patients with PCLs, including 16 cases of IPMN and 1 case of mucinous cystadenoma. Follow-up results at 12 months demonstrated CR in 11 of 17 PCLs (65%), with 1 case achieving a >50% reduction in diameter, yielding a significant response rate of 71%. Additionally, EUS follow-up revealed CR of mural nodules in all 12/12 (100%) evaluable patients. Furthermore, Barthet et al.^[54]^ conducted a long-term follow-up to investigate the prognosis of EUS-RFA, and among the 17 initial participants, 2 were excluded due to deaths unrelated to the procedure. In the remaining 15 patients at the end of follow-up (42.6 months), CR was observed in 6 cases (40%), and a >50% reduction in cyst diameter was achieved in 4 cases (26.6%). Notably, mural nodules, present in 10 patients at baseline, were completely absent during follow-up. These findings suggest stable therapeutic efficacy of EUS-RFA for PCLs. Similarly, a retrospective analysis involving 10 patients with IPMNs accompanied by mural nodules reported that all patients exhibited CR of the enhanced mural nodules following EUS-RFA, demonstrating effective ablation.^[56]^ Furthermore, Younis et al.^[55]^ conducted a small prospective single-center study involving 4 IPMN patients exhibiting worrisome features and 1 MCN patient. The results demonstrated that, among the 5 PCL patients, complete radiologic response was achieved in 3 cases (60%) and PR in 1 case (20%), suggesting that EUS-RFA is a technically feasible and well-tolerated therapeutic approach for selected patients with premalignant PCLs.

In a retrospective study on SCNs, Oh et al.^[13]^ enrolled 13 patients with microcystic SCNs demonstrating a honeycomb appearance. Among them, 7 patients underwent a single session of EUS-RFA, whereas 6 patients received 2 sessions. Postoperative follow-up results revealed that the median cyst volume decreased from 37.82 to 10.95 mL at the end of the follow-up. Radiographic PR was achieved in 8 patients (61.5%), with only one case reporting postoperative self-limiting abdominal pain. Notably, CR was not observed in any patient, which may be associated with the multiple septations within microcystic SCNs influencing the therapeutic response. Importantly, despite the absence of complete cyst resolution, all patients exhibited symptomatic improvement following SCN size reduction, suggesting that EUS-RFA represents a viable treatment modality for microcystic SCNs with an acceptable adverse event profile. However, long-term follow-up remains necessary to evaluate its sustained therapeutic efficacy.

In summary, EUS-RFA is a feasible and well-tolerated treatment, but patient selection for PCLs is still debated. Ablation decisions should be discussed within a multidisciplinary team, considering factors such as the malignant potential of PCLs, life expectancy, and postoperative risks. Further rigorous studies and follow-up are warranted to explore and evaluate the long-term therapeutic outcomes of EUS-RFA.

## CONCLUSION

Striking a balance between the risks of resecting benign lesions and the inadequate treatment of malignant cysts, as well as weighing the risks and costs of ongoing invasive and noninvasive surveillance, remains a complex challenge for the management of PCLs. According to current American Gastroenterological Association guidelines, there is insufficient evidence to support the routine use of EUS-PCA.^[8,9]^ Nevertheless, recent high-quality studies^[10,17]^ have demonstrated the unique advantages of EUS-PCA over surveillance or surgery, suggesting that EUS-PCA may become an appropriate treatment option for certain specific populations. Moreover, EUS-PCA may serve as an effective alternative for patients who decline or are ineligible for surgery.^[1]^

Ethanol demonstrates favorable safety and efficacy for PCL treatment but shows limitations in managing malignancies such as IPMNs. Lauromacrogol offers significant advantages in reducing perioperative pain due to its mild anesthetic properties. The combination of ethanol and paclitaxel exhibits superior efficacy to ethanol monotherapy and demonstrates the ability to eradicate oncogenic driver mutations such as K-ras, thereby mitigating the risk of malignant transformation. Furthermore, alcohol-free protocols have been proposed to minimize complications with similar efficacy. EUS-RFA represents a technically feasible and well-tolerated modality, particularly effective for PCLs with high malignant potential, such as those containing mural nodules.

Further prospective studies with longer follow-up are warranted to explore and evaluate the therapeutic efficacy and safety of EUS-PCA.

## Acknowledgment

None.

## Source of Funding

This study was funded by the General Program of the National Natural Science Foundation of China, no. 82370655, and the Young Scientists Fund of the National Natural Science Foundation of China, no. 82300734.

## Ethical Approval

Not applicable.

## Informed Consent

Not applicable.

## Conflicts of Interest

Zhendong Jin is an associate editor of the journal. This article was subject to the journal’s standard procedures, with peer review handled independently of the editor and his research group. The authors declare that they have no financial conflict of interest with regard to the content of this report.

## Author Contributions

Z. Jin, H. Huang, and L. Sun contributed to the study conception and design. Material preparation and data collection were performed by L. Peng and Y. Sun. S. Li created the tables. The first draft of the manuscript was written by Mengruo Jiang, and all authors commented on previous versions of the manuscript. Z. Li, Z. Jin, and H. Huang revised the manuscript. All authors read and approved the final manuscript.

## Data Availability Statement

Data sharing is not applicable to this article, as no new data were created or analyzed in this study.
